# *Salmonella enterica* I 4,[5],12:i:- Associated with Lesions Typical of Swine Enteric *Salmonellosis*

**DOI:** 10.3201/eid2507.181453

**Published:** 2019-07

**Authors:** Bailey L. Arruda, Eric R. Burrough, Kent J. Schwartz

**Affiliations:** Iowa State University, Ames, Iowa, USA

**Keywords:** Salmonella, *Salmonella enterica* I 4,[5],12:i-, bacteria, serotype, swine, lesions, swine enteric salmonellosis, enteric infections, zoonoses

## Abstract

*Salmonella enterica* serotype I 4,[5],12:i:- has been increasingly isolated from swine. However, its pathogenic potential is not well characterized. Analysis of swine cases confirmed a strong positive association between isolation of I 4,[5],12:i:- and lesions of enteric salmonellosis and suggested a similar pathogenic potential as that for *Salmonella* Typhimurium.

Over the past decade, *Salmonella enterica* serotype I 4,[5],12:i:- has emerged as a major public health threat in Europe (*1*) and the United States (*2*). As a monophasic variant of *Salmonella* Typhimurium, *Salmonella* I 4,[5],12:i:- was rarely identified before the mid-1990s (*3*). However, it has now been detected in cattle (*4*), poultry (*5*), and swine (*4**–**6*), and several human disease outbreaks associated with contaminated pork products have occurred (*7**–**10*).

Salmonellosis is also a major disease concern in swine. *Salmonella* Typhimurium has been considered the most common cause of enteric salmonellosis in swine (*11*). Recent reports from 2 of the largest veterinary diagnostic laboratories in the United States showed that there was a noted increase in the percentage of isolates of *Salmonella* I 4,[5],12:i:- (*4**,**6*).

Despite the apparent increase in the isolation of *Salmonella* I 4,[5],12:i:- from swine and pork products, there is currently limited information regarding the pathogenicity of this serotype in swine. Accordingly, this study assessed the pathogenic potential of *Salmonella* I 4,[5],12:i:- through the evaluation of microscopic intestinal lesions in swine enteric cases from which *Salmonella* I 4,[5],12:i:- was isolated compared with similar cases from which *Salmonella* I 4,[5],12:i:- was not isolated.

## The Study

The Iowa State University–Veterinary Diagnostic Laboratory (ISU-VDL) is a National Animal Health Laboratory Network accredited laboratory that receives >75,000 case submissions annually, of which most are derived from swine production systems located throughout the United States. Histopathologic analysis is performed by veterinary diagnostic pathologists on ≈10,000 cases from swine per year. The ISU-VDL laboratory information management system provided the initial dataset for this analysis.

To determine whether isolation of *Salmonella* I 4,[5],12:i:- from samples submitted from pigs was associated with microscopic lesions consistent with enteric salmonellosis, we compared cases from which *Salmonella* I 4,[5],12:i:- was isolated with cases from which neither *Salmonella* I 4,[5],12:i:- nor *Salmonella* Typhimurium were isolated; these samples were collected during July 2016–December 2017. We also reviewed cases from which *Salmonella* Typhimurium or 1 of 3 *Salmonella* serogroup B serovars (*Salmonella* Derby, *Salmonella* Agona, and *Salmonella* Heidelberg) were isolated to determine the potential comparative pathogenicity of Salmonella I 4,[5],12:i:-. All of these cases met the following qualifying criteria: animals were 3–13 weeks of age, a *Salmonella* serovar as outlined above was isolated by direct culture performed on enteric tissues, and histopathologic analysis was performed on the large intestine. To determine the association between the presence of *Salmonella* I 4,[5],12:i:- and lesions consistent with enteric salmonellosis, we also reviewed 40 additional cases that met the previously stated inclusion criteria but from which neither *Salmonella* I 4,[5],12:i:- nor *Salmonella* Typhimurium were isolated; we randomly selected these cases from an Excel (Microsoft, https://www.microsoft.com) data file by using the RAND() function.

Enteric samples submitted for isolation of *Salmonella* were routinely processed and reported (Appendix). Microscopic lesions deemed compatible with salmonellosis in sections of the large intestine included erosion; ulceration; neutrophilic infiltration; crypt ectasia, crypt elongation, or both with associated neutrophilic exudation; goblet cell loss; luminal accumulation of neutrophils and fibrin; and submucosal accumulation of lymphocytes and macrophages. We used JMP Pro 14 (SAS Institute, https://www.sas.com) to perform all analyses. We used the Pearson χ^2^ test and odds ratios to determine the association between isolation of *Salmonella* I 4,[5],12:i:- and pathologic diagnosis of enteric salmonellosis. We considered a p value <0.05 significant.

We isolated *Salmonella* I 4,[5],12:i:- from 138 porcine cases that met all of the qualifying criteria. We isolated *Salmonella* Typhimurium from 18 cases, and other potentially lesser pathogenic *Salmonella* serogroup B serovars, including *Salmonella* Derby, Agona, and Heidelberg, from 35 cases.

A review of case data for clinical submissions to the ISU-VDL confirmed a statistically significant positive association between histologic lesions consistent with enteric salmonellosis and isolation of *Salmonella* I 4,[5],12:i:- (odds ratio 10.53, 95% CI 4.45–24.88; p<0.0001) (Table). We confirmed compatible histologic lesions of salmonellosis (Figure) for 100 (72%) of 138 cases from which *Salmonella* I 4,[5],12:i:- was isolated (Appendix Table 1). Histologic lesions consistent with enteric salmonellosis from which neither *Salmonella* I 4,[5],12:i:- nor *Salmonella* Typhimurium were isolated were observed for 8 (20%) of 40 cases (Appendix Table 2).

**Table:** Diagnostic cases with and without colitis in swine and isolation of *Salmonella enterica* I 4,[5],12:i:-*

I 4,[5],12:i:- isolated	Colitis lesion	
	No	Yes
No	32	8
Yes	38	100

*Odds ratio 10.5 (95% CI 4.45–24.88); p<0.0001 by Pearson χ^2^ test.

**Figure:**
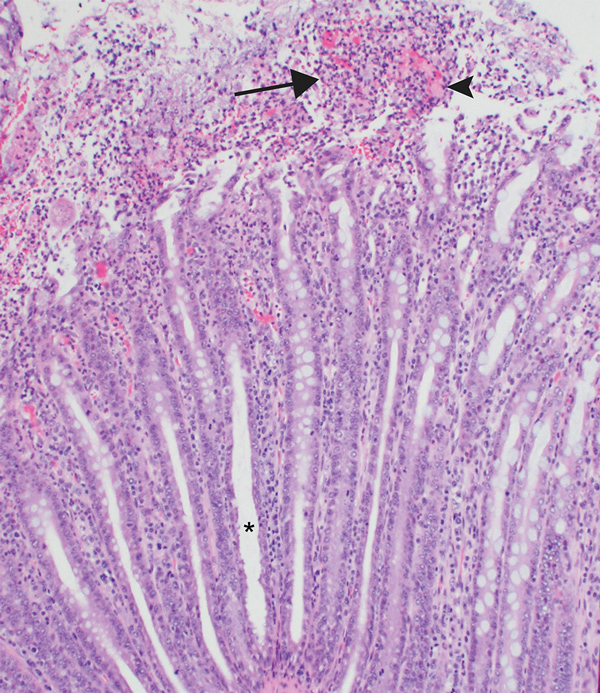
Section of large intestine from a pig infected with *Salmonella enterica* subsp. *enterica* serotype I 4,[5],12:i:-. Asterisk indicates crypt elongation and goblet cell loss, arrow indicates abundant degenerate neutrophils in the lumen, and arrowhead indicates abundant fibrin in the lumen. Hematoxylin and eosin stain; original magnification ×100.

*Salmonella* was isolated from 6 of the 40 cases, 3 of which had lesions consistent with enteric salmonellosis. We confirmed compatible histologic lesions of salmonellosis in 17 (94%) of 18 cases in which *Salmonella* Typhimurium was isolated and 11 (31%) of 35 cases in which another serogroup B *Salmonella* was isolated. Other agents of enteric disease that were concurrently detected in some cases included rotaviruses, coronaviruses, coccidians, and hemolytic *Escherichia coli*. However, none of these agents caused lesions consistent with those used to define salmonellosis in this report (Appendix Tables 1, 2).

## Conclusions

During an 18-month period and using the same qualifying criteria for cases, we identified *Salmonella* I 4,[5],12:i:- from 138 swine cases. However, *Salmonella* Typhimurium was isolated from only 18 cases and another serogroup B *Salmonella* as specified above was isolated from only 35 cases. This finding represents nearly an 8-fold increase in isolation of *Salmonella* I 4,[5],12:i:- compared with *Salmonella* Typhimurium and is in concordance with findings of others who have identified an increase in isolation of *Salmonella* I 4,[5],12:i:- from swine samples submitted to veterinary diagnostic laboratories (*4**,**6*).

A major aspect of *Salmonella* epidemiology is the variation in prevalence of serotypes or phage types over time in human and animal populations. The catalysts of such changes remain elusive (*3*). Isolation of *Salmonella* I 4,[5],12:i:- was rarely reported before 1993 (*3*), but this serotype has now become predominant in human clinical cases and has been isolated from food products, including pork, on different continents (*3*).

Although increased isolation of *Salmonella* I 4,[5],12:i:- from swine samples has been documented, the pathogenic potential of this serovar in pigs had not been reported. In this study, we demonstrate a strong positive association between histologic lesions consistent with enteric salmonellosis and isolation of *Salmonella* I 4,[5],12:i:-. In most cases from which *Salmonella* I 4,[5],12:i:- or *Salmonella* Typhimurium were isolated, the severity of histologic lesions was similar. However, the percentage of cases in which histologic lesions consistent with enteric salmonellosis were present was lower for cases from which *Salmonella* I 4,[5],12:i:- (72%) was isolated than for cases from which *Salmonella* Typhimurium (94%) was isolated. Based on these data, we believe that *Salmonella* I 4,[5],12:i:- is a likely cause of enteric salmonellosis that has a similar or perhaps slightly lower pathogenic potential in swine than *Salmonella* Typhimurium. Pathogenicity studies are needed to further characterize the pathogenic potential and fitness of *Salmonella* I 4,[5],12:i:- compared with that of *Salmonella* Typhimurium in swine.

We suspect that *Salmonella* I 4,[5],12:i:- has evolutionary advantages that have resulted in its predominance as one of the most common *Salmonella* serotypes responsible for swine enteric salmonellosis. Accordingly, it is essential to determine the putative attributes that facilitate its rapid spread and ecologic success. Specifically, antimicrobial drug resistance genes and genes that encode resistance to heavy metal micronutrients should be evaluated, given their current and common use in US swine production.

Appendix Additional information on *Salmonella enterica* I 4,[5],12:i:- associated with lesions typical of swine enteric salmonellosis.
