# Supplementary material for: Salmonella enterica I 4,[5],12:i:- Associated with Lesions Typical of Swine Enteric Salmonellosis
Source: Emerg Infect Dis. 2019 Jul;25(7):1377–9. doi: 10.3201/eid2507.181453 (PMC6590737; doi:10.3201/eid2507.181453)
Supplement: Appendix — Additional information on Salmonella enterica I 4,[5],12:i:- associated with lesions typical of swine enteric salmonellosis. [file 18-1453-Techapp-s1.pdf]

Article DOI: https://doi.org/10.3201/eid2507.181453

Salmonella enterica I 4,[5],12:i:- Associated with Lesions Typical of Swine Enteric Salmonellosis

Appendix

Enteric samples submitted for bacterial isolation were routinely processed as follows. Clinical samples were directly inoculated onto solid media (sheep blood agar, brilliant green agar, and tergitol-7 agar) and incubated for 18–24 hours at 35°C ± 2°C. When colonies with a phenotype consistent with *Salmonella* were detected and confirmed at the genus level by using matrix-assisted laser desorption/ionization time-of-flight mass spectrometry, 1 CFU belonging to each phenotype identified in each processed sample was analyzed to determine its serogroup by using either BD Difco Salmonella O Antisera for Groups C3, E, and Poly (Becton Dickinson, https://www.bd.com) or Statens Serum Institut O:4, O:7, O:8, or O:9 O group rabbit antiserum (Statens Serum Institut, https://en.ssi.dk) for group B, C1, C2, or D, respectively. Bacterial isolates were then subcultured and submitted for serotyping at the National Veterinary Services Laboratory (Ames, IA, USA).

Appendix Table 1. Diagnostic data for 138 swine cases from which *Salmonella enterica* subsp. *enterica* serotype I 4,[5],12:i:- was isolated*

Case	State	Age, wk	Bacterial culture†	Coronavirus‡	Rotavirus§	Diagnosis¶	Group
1	IA	6.0	Hemolytic *Escherichia coli*; NP	Pos	Pos	Viral enteritis	N
2	IA	6.0	Hemolytic *E. coli*	NA	NA	No etiologic diagnosis	N
3	IA	6.0	Hemolytic *E. coli*	NA	NA	Colibacillosis	N
4	IA	6.0	Hemolytic *E. coli*	NA	NA	No etiologic diagnosis	N
5	IA	5.0	*Salmonella* I 4,[5],12:i:-	NA	NA	Bacterial septicemia	N
6	IA	5.5	Hemolytic *E. coli*; NP	NA	NA	Colibacillosis	N
7	IA	6.0	*Salmonella* I 4,[5],12:i:-	Neg	NA	No etiologic diagnosis	N
8	IA	10.0	*Salmonella* I 4,[5],12:i:-	Neg	NA	No etiologic diagnosis	N
9	IA	5.0	*Salmonella* I 4,[5],12:i:-	Pos	NA	Viral enteritis	N
10	IA	11.0	*Salmonella* I 4,[5],12:i:-	Pos	Neg	No etiologic diagnosis	N
11	NA	10.0	*Salmonella* I 4,[5],12:i:-	Neg	NA	Salmonellosis	Y
12	IA	6.0	Hemolytic *E. coli*	NA	NA	Salmonellosis; colibacillosis	Y
13	NA	8.5	*Salmonella* I 4,[5],12:i:-	Neg	Pos	Salmonellosis	Y
14	NC	12.0	*Salmonella* I 4,[5],12:i:-	Neg	NA	Salmonellosis	Y
15	IA	8.0	*Salmonella* I 4,[5],12:i:-	Neg	Pos	Salmonellosis	Y
16	MN	6.0	Hemolytic *E. coli*	NA	NA	Salmonellosis; colibacillosis	Y
17	IA	7.0	*Salmonella* I 4,[5],12:i:-	Neg	NA	Salmonellosis	Y
18	MO	8.0	Hemolytic *E. coli*; NP	NA	NA	Salmonellosis	Y
19	IA	7.0	*Salmonella* I 4,[5],12:i:-	Pos	NA	Salmonellosis	Y
20	IA	7.0	Hemolytic *E. coli*	Neg	Pos	Salmonellosis	Y
21	IL	6.0	Hemolytic *E. coli*	NA	NA	Salmonellosis	Y

Case	State	Age, wk	Bacterial culture†	Coronavirus‡	Rotavirus§	Diagnosis¶	Group
22	IA	9.0	*Salmonella* I 4,[5],12:i:-	Neg	NA	Salmonellosis	Y
23	MO	8.0	Hemolytic *E. coli*	NA	NA	Salmonellosis	Y
24	IA	7.0	Hemolytic *E. coli*	NA	NA	Salmonellosis; colibacillosis	Y
25	MD	4.0	*Salmonella* I 4,[5],12:i:-	NA	NA	Salmonellosis	Y
26	NA	6.0	*Salmonella* I 4,[5],12:i:-	Neg	NA	Salmonellosis	Y
27	MN	6.0	*Salmonella* I 4,[5],12:i:-	Neg	NA	Salmonellosis	Y
28	MN	12.0	*Salmonella* I 4,[5],12:i:-	Pos	NA	Salmonellosis	Y
29	NA	5.5	*Salmonella* I 4,[5],12:i:-	NA	NA	Salmonellosis	Y
30	IA	6.0	*Salmonella* I 4,[5],12:i:-	NA	NA	Salmonellosis	Y
31	IA	8.0	*Salmonella* I 4,[5],12:i:-	NA	NA	Salmonellosis	Y
32	MN	6.5	*Salmonella* I 4,[5],12:i:-	NA	NA	Salmonellosis	Y
33	IA	7.0	Hemolytic *E. coli*	NA	NA	Salmonellosis; colibacillosis	Y
34	IA	6.0	Hemolytic *E. coli*	Pos	Pos	Salmonellosis; colibacillosis	Y
35	IA	6.0	Hemolytic *E. coli*	NA	Neg	Salmonellosis	Y
36	MO	9.0	Hemolytic *E. coli*	NA	NA	Salmonellosis	Y
37	IA	7.0	*Salmonella* I 4,[5],12:i:-	NA	NA	Salmonellosis	Y
38	IA	8.0	*Salmonella* I 4,[5],12:i:-	NA	NA	Salmonellosis	Y
39	MO	7.0	Hemolytic *E. coli*	NA	Neg	Salmonellosis	Y
40	IA	8.0	*Salmonella* I 4,[5],12:i:-	NA	Neg	Salmonellosis	Y
41	IA	5.5	Hemolytic *E. coli*; P	NA	Pos	Salmonellosis	Y
42	NA	7.0	Hemolytic *E. coli*; P	NA	NA	Salmonellosis; colibacillosis	Y
43	IL	6.0	*Salmonella* I 4,[5],12:i:-	Neg	NA	Salmonellosis	Y
44	NA	6.0	*Salmonella* I 4,[5],12:i:-	Neg	Pos	Salmonellosis	Y
45	MN	5.5	Hemolytic *E. coli*	Pos	Pos	Salmonellosis	Y
46	IA	7.0	*Salmonella* I 4,[5],12:i:-	Pos	Neg	Salmonellosis	Y
47	PA	8.0	Hemolytic *E. coli*; NP	NA	NA	Salmonellosis	Y
48	IA	6.0	Hemolytic *E. coli*	Neg	Pos	Colibacillosis	N
49	NC	11.0	*Salmonella* I 4,[5],12:i:-	NA	NA	No etiologic diagnosis	N
50	OK	6.0	*Salmonella* I 4,[5],12:i:-	NA	NA	No etiologic diagnosis	N
51	IA	7.0	*Salmonella* I 4,[5],12:i:-	NA	Neg	Salmonellosis	Y
52	IA	10.0	*Salmonella* I 4,[5],12:i:-	Neg	Pos	Salmonellosis	Y
53	IA	8.0	*Salmonella* I 4,[5],12:i:-	NA	Pos	Salmonellosis	Y
54	NA	6.5	Hemolytic *E. coli*; P	Neg	NA	Colibacillosis	N
55	WI	8.0	*Salmonella* I 4,[5],12:i:-	NA	NA	No etiologic diagnosis	N
56	IA	9.0	*Salmonella* I 4,[5],12:i:-	NA	NA	Salmonellosis	Y
57	IA	8.0	*Salmonella* I 4,[5],12:i:-	Neg	NA	Salmonellosis	Y
58	IA	7.0	*Salmonella* I 4,[5],12:i:-	NA	NA	Salmonellosis	Y
59	NA	7.5	*Salmonella* I 4,[5],12:i:-	NA	Pos	Salmonellosis	Y
60	NC	4.0	*Salmonella* I 4,[5],12:i:-	NA	NA	No etiologic diagnosis	N
61	OH	8.0	Hemolytic *E. coli*; NP	NA	NA	Salmonellosis	Y
62	MO	7.0	Hemolytic *E. coli*	Neg	Pos	Salmonellosis	Y
63	IA	4.0	Hemolytic *E. coli*; P	Neg	Pos	No etiologic diagnosis	N
64	IA	7.0	Hemolytic *E. coli*	Neg	Pos	Salmonellosis	Y
65	MN	5.0	Hemolytic *E. coli*; P	NA	NA	No etiologic diagnosis	N
66	IA	6.0	*Salmonella* I 4,[5],12:i:-	NA	Pos	Viral enteritis	N
67	IA	7.0	Hemolytic *E. coli*	NA	NA	Salmonellosis	Y
68	IA	7.0	Hemolytic *E. coli*	NA	Pos	Salmonellosis	Y
69	NC	6.0	Hemolytic *E. coli*	NA	NA	Salmonellosis	Y
70	IA	7.0	*Salmonella* I 4,[5],12:i:-	NA	NA	No etiologic diagnosis	Y
71	IA	5.0	Hemolytic *E. coli*; P	NA	NA	No etiologic diagnosis	Y
72	IA	10.0	Hemolytic *E. coli*	Pos	Pos	Salmonellosis	Y
73	NC	6.0	Hemolytic *E. coli*	NA	NA	Salmonellosis	Y
74	IA	13.0	*Salmonella* I 4,[5],12:i:-	Pos	NA	Salmonellosis	Y
75	NC	10.0	Hemolytic *E. coli*	Neg	NA	Salmonellosis	Y
76	IA	10.0	*Salmonella* I 4,[5],12:i:-	NA	NA	No etiologic diagnosis	N
77	IA	8.0	Hemolytic *E. coli*	Neg	Neg	Salmonellosis	Y
78	IA	5.5	Hemolytic *E. coli*	Pos	NA	Salmonellosis	Y
79	IA	7.0	Hemolytic *E. coli*	Neg	Pos	No etiologic diagnosis	N
80	NA	13.0	Hemolytic *E. coli*	Neg	NA	No etiologic diagnosis	N
81	NA	8.0	*Salmonella* I 4,[5],12:i:-	Neg	NA	Salmonellosis	Y
82	PA	8.0	*Salmonella* I 4,[5],12:i:-	NA	NA	Salmonellosis	Y
83	IA	8.0	*Salmonella* I 4,[5],12:i:-	Neg	Pos	Salmonellosis	Y
84	CA	10.0	Hemolytic *E. coli*; NP	NA	NA	Porcine proliferative enteritis	N
85	IL	6.0	*Salmonella* I 4,[5],12:i:-	Neg#	Pos	Salmonellosis	Y

Case	State	Age, wk	Bacterial culture†	Coronavirus‡	Rotavirus§	Diagnosis¶	Group
86	NA	5.0	*Salmonella* I 4,[5],12:i:-	Neg	Pos	Salmonellosis	Y
87	MO	7.0	*Salmonella* I 4,[5],12:i:-	NA	NA	No etiologic diagnosis	N
88	IA	8.0	Hemolytic *E. coli*	NA	NA	No etiologic diagnosis	Y
89	AK	5.0	*Salmonella* I 4,[5],12:i:-	Neg	Pos	Salmonellosis	Y
90	IL	6.0	*Salmonella* I 4,[5],12:i:-	NA	NA	No etiologic diagnosis	N
91	NA	8.0	*Salmonella* I 4,[5],12:i:-	NA	NA	Salmonellosis	Y
92	IA	8.0	*Salmonella* I 4,[5],12:i:-	NA	NA	Salmonellosis	Y
93	IA	6.0	*Salmonella* I 4,[5],12:i:-	NA	NA	Salmonellosis	Y
94	NA	10.0	Hemolytic *E. coli*	NA	NA	Salmonellosis	Y
95	NA	6.5	*Salmonella* I 4,[5],12:i:-	NA	NA	Salmonellosis	Y
96	MN	6.0	*Salmonella* I 4,[5],12:i:-	NA	NA	Salmonellosis	Y
97	NC	6.0	Hemolytic *E. coli*	Neg	Pos	No etiologic diagnosis	N
98	IA	7.0	*Salmonella* I 4,[5],12:i:-	Neg	Pos	Salmonellosis	Y
99	NA	8.0	*Salmonella* I 4,[5],12:i:-	NA	NA	Salmonellosis	Y
100	NA	7.0	*Salmonella* I 4,[5],12:i:-	NA	NA	Salmonellosis	Y
101	OH	6.0	Hemolytic *E. coli*	Neg#	NA	Salmonellosis	Y
102	IA	4.5	*Salmonella* I 4,[5],12:i:-	Neg#	Pos	Salmonellosis	Y
103	IA	8.0	*Salmonella* I 4,[5],12:i:-	Neg#	Pos	Salmonellosis	Y
104	IA	7.0	Hemolytic *E. coli*; P	Neg	NA	Salmonellosis	Y
105	IA	13.0	*Salmonella* I 4,[5],12:i:-	Pos	NA	Salmonellosis	Y
106	NA	8.0	Hemolytic *E. coli*	NA	NA	Salmonellosis	Y
107	KY	6.0	Hemolytic *E. coli*	NA	NA	Salmonellosis	Y
108	IA	8.0	Hemolytic *E. coli*; NP	NA	NA	Salmonellosis	Y
109	IA	12.0	*Salmonella* I 4,[5],12:i:-	NA	NA	Salmonellosis	Y
110	IA	6.0	Hemolytic *E. coli*	Neg	Pos	Colibacillosis	N
111	MN	12.0	Hemolytic *E. coli*	NA	NA	No etiologic diagnosis	N
112	NA	6.8	*Salmonella* I 4,[5],12:i:-	Neg	Neg	Salmonellosis	Y
113	IA	8.0	Hemolytic *E. coli*; NP	NA	NA	Salmonellosis	Y
114	AL	10.0	*Salmonella* I 4,[5],12:i:-	NA	NA	Salmonellosis	Y
115	IA	7.0	*Salmonella* I 4,[5],12:i:-	Neg	Pos	Salmonellosis	Y
116	IA	7.0	Hemolytic *E. coli*	NA	NA	No etiologic diagnosis	N
117	IA	8.0	Hemolytic *E. coli*	NA	Pos	No etiologic diagnosis	N
118	IA	8.0	*Salmonella* I 4,[5],12:i:-	NA	NA	No etiologic diagnosis	Y
119	NC	11.0	*Salmonella* I 4,[5],12:i:-	Neg#	Pos	Salmonellosis	Y
120	IA	13.0	*Salmonella* I 4,[5],12:i:-	NA	NA	Porcine proliferative enteritis	N
121	IA	6.5	*Salmonella* I 4,[5],12:i:-	Neg	Pos	Salmonellosis	Y
122	NA	5.5	Hemolytic *E. coli*; NP	NA	NA	No etiologic diagnosis	N
123	NA	8.0	*Salmonella* I 4,[5],12:i:-	NA	NA	Salmonellosis	Y
124	IA	10.0	Hemolytic *E. coli*	NA	NA	Salmonellosis	Y
125	MN	12.0	*Salmonella* I 4,[5],12:i:-	NA	NA	Salmonellosis	Y
126	OK	5.0	Hemolytic *E. coli*	NA	NA	Colibacillosis	N
127	NC	6.0	Hemolytic *E. coli*	NA	NA	Salmonellosis	Y
128	NC	8.0	*Salmonella* I 4,[5],12:i:-	NA	NA	No etiologic diagnosis	N
129	NC	5.0	*Salmonella* I 4,[5],12:i:-	Pos	Pos	Salmonellosis	Y
130	IA	7.0	*Salmonella* I 4,[5],12:i:-	NA	NA	Salmonellosis	Y
131	IA	5.0	*Salmonella* I 4,[5],12:i:-	Neg	Pos	Viral enteritis; dysbiosis	N
132	NA	5.0	*Salmonella* I 4,[5],12:i:-	NA	Pos	Viral enteritis	N
133	NC	5.0	*Salmonella* I 4,[5],12:i:-	NA	Pos	Salmonellosis	Y
134	IA	6.0	*Salmonella* I 4,[5],12:i:-	Neg	Pos	Salmonellosis	Y
135	NC	7.0	Hemolytic *E. coli*; P	NA	NA	Salmonellosis	Y
136	IA	5.0	Hemolytic *E. coli*	Neg	Pos	No etiologic diagnosis	N
137	NC	5.0	*Salmonella* I 4,[5],12:i:-	Neg	Pos	Salmonellosis	Y
138	IA	6.0	*Salmonella* I 4,[5],12:i:-	NA	NA	No etiologic diagnosis	N

*N, no lesions consistent with enteric salmonellosis; NA, not available; Neg, negative; Pos, positive; P, pathogenic *E. coli* identified by genotyping PCR; NP, nonpathogenic *E. coli* identified by genotyping PCR; Y, lesions consistent with enteric salmonellosis.
†Bacterial isolation of other potential enteric pathogens of swine. If only *Salmonella* I 4,[5],12:i:- was isolated, *Salmonella* I 4,[5],12:i:- is listed.
‡Porcine epidemic diarrhea virus and porcine deltacoronavirus PCR results.
§Porcine rotavirus group A, B, and C PCR results.
¶Laboratory diagnosis based on clinical signs, gross and histologic lesions, and ancillary diagnostics.
#Transmissible gastroenteritis virus PCR results were also negative.

Appendix Table 2. Diagnostic data for 40 swine cases from which *Salmonella enterica* subsp. *enterica* serotype I 4,[5],12:i:- was not isolated*

Case	State	Age, wk	Bacterial culture†	Coronavirus‡	Rotavirus§	Diagnosis¶	Group
1	IA	4.0	*Salmonella* enterica Manhattan	NA	NA	Coccidiosis	N
2	IA	3.0	Hemolytic *Escherichia coli*	Neg	Pos	Colibacillosis	N
3	IA	6.0	Hemolytic *E. coli*; P	NA	NA	Colibacillosis	N
4	IA	6.0	Hemolytic *E. coli*	NA	NA	Colibacillosis	N
5	IA	3.0	Hemolytic *E. coli*	NA	NA	Colitis, fibrinopurulent	Y
6	NC	3.0	Hemolytic *E. coli*	NA	NA	Colitis, fibrinopurulent	Y
7	OH	4.0	*Salmonella* Johannesburg	NA	NA	Dysbiosis	N
8	IL	3.0	Hemolytic *E. coli*	NA	NA	Dysbiosis	N
9	MN	4.0	Hemolytic *E. coli*	NA	NA	Dysbiosis	N
10	NA	4.5	No major growth	NA	NA	Salmonellosis; dysbiosis	Y
11	IA	13.0	No major growth	NA	NA	Hemorrhagic bowel disease	N
12	IA	6.0	Hemolytic *E. coli*	NA	NA	No etiologic diagnosis	N
13	IA	4.0	Hemolytic *E. coli*; *Salmonella* Infantis	Neg#	NA	Salmonellosis	Y
14	NA	10.0	No major growth	NA	NA	No etiologic diagnosis	N
15	OH	10.0	Hemolytic *E. coli*	NA	NA	No etiologic diagnosis	N
16	IA	12.0	No major growth	NA	NA	No etiologic diagnosis	N
17	NA	6.5	Hemolytic *E. coli*	NA	NA	No etiologic diagnosis	N
18	IA	8.0	Hemolytic *E. coli*	NA	NA	No etiologic diagnosis	N
19	OH	12.0	No major growth	NA	NA	No etiologic diagnosis	N
20	NA	10.0	Hemolytic *E. coli*	NA	NA	No etiologic diagnosis	N
21	NA	6.0	No major growth	NA	NA	No etiologic diagnosis	N
22	IA	9.0	Hemolytic *E. coli*	Neg	NA	No etiologic diagnosis	N
23	IA	3.5	No major growth	NA	NA	No etiologic diagnosis	N
24	IN	7.0	Smooth *E. coli*	NA	NA	No etiologic diagnosis	N
25	OH	3.0	No major growth	Neg	Pos	No etiologic diagnosis	N
26	IA	10.0	Hemolytic *E. coli*	NA	NA	No etiologic diagnosis	N
27	IA	4.0	No major growth	NA	NA	No etiologic diagnosis	N
28	IA	4.0	No major growth	Neg	Pos	No etiologic diagnosis	N
29	MO	7.0	No major growth	NA	NA	No etiologic diagnosis	N
30	IA	3.5	No major growth	NA	NA	No etiologic diagnosis	N
31	NE	6.0	No major growth	NA	NA	No etiologic diagnosis	N
32	NA	5.0	Hemolytic *E. coli*; NP	NA	NA	No etiologic diagnosis	N
33	IA	8.0	No major growth	NA	NA	Salmonellosis	Y
34	NA	4.0	Hemolytic *E. coli*; *Salmonella* Ohio	NA	NA	Salmonellosis	Y
35	IA	9.0	*Salmonella* Muenchen	Neg#	NA	Salmonellosis	Y
36	IA	5.0	No major growth	NA	NA	Viral enteritis	N
37	NA	5.0	*Salmonella* Derby	NA	Pos	Viral enteritis	N
38	NC	4.0	Hemolytic *E. coli*	Neg	Pos	Viral enteritis; colibacillosis	N
39	IA	4.0	Hemolytic *E. coli*; P	Pos	NA	Viral enteritis; colibacillosis	N
40	NC	4.0	Hemolytic *E. coli*; P	NA	NA	Viral enteritis; colibacillosis; colitis	Y

*N, no lesions consistent with enteric salmonellosis; NA, not available; Neg, negative; Pos, positive; P, pathogenic *E. coli* identified by genotyping PCR; NP, nonpathogenic *E. coli* identified by genotyping PCR; Y, lesions consistent with enteric salmonellosis.
†Bacterial isolation of other potential enteric pathogens of swine.
‡Porcine epidemic diarrhea virus and porcine deltacoronavirus PCR results.
§Porcine rotavirus group A, B, and C PCR results.
¶Laboratory diagnosis based on clinical signs, gross and histologic lesions, and ancillary diagnostics.
#Transmissible gastroenteritis virus PCR results were also negative.
